# Prevalence of ophthalmological manifestations in pediatric and adolescent populations with Down syndrome: a systematic review of the literature

**DOI:** 10.1186/s13643-022-01940-5

**Published:** 2022-04-22

**Authors:** Juliana Muñoz-Ortiz, Jesús David Charry-Sánchez, Isabella Bechara-Arango, Mariana Blanco-Becerra, Claudia Talero-Gutiérrez, Marcela Gomez-Suarez, Alejandra de-la-Torre

**Affiliations:** 1 Neuroscience Research Group (NEUROS), NeuroVitae Center, Escuela de Medicina y Ciencias de la Salud, Universidad del Rosario, Bogotá, Colombia; 2 Grupo de investigación Escuela Barraquer, Escuela Superior de Oftalmología del Instituto Barraquer de America, Bogotá, Colombia; 3 Instituto de Investigaciones, Fundación Universitaria de Ciencias de la Salud (FUCS), Bogotá, Colombia

**Keywords:** Down syndrome, Children, Ocular findings, Eye manifestations, Prevalence, Systematic review

## Abstract

**Background:**

Down syndrome (DS) is a chromosomal anomaly that is characterized by an extra chromosome 21. Ophthalmological manifestations have a high prevalence in patients with DS.

**Purpose:**

To review the scientific evidence and estimate the prevalence of ophthalmological manifestations in the pediatric population with DS.

**Data sources:**

Electronic databases including MEDLINE, Cochrane Library, EMBASE, ScienceDirect, and LILACS.

**Study eligibility criteria:**

Published observational studies with available and original data were included. Articles were excluded if the study design was a review, letter to the editor, case report, case series, or systematic review and if the subjects had ophthalmological manifestations secondary to other conditions.

**Participants and interventions:**

Pediatric and adolescent population with DS and with ophthalmological evaluation.

**Study appraisal and synthesis methods:**

A data collection form was designed in Excel. Five reviewers extracted relevant details about the design and results of each study. The quality of the studies was assessed by applying the tools for systematic reviews of prevalence and incidence from the Joanna Briggs Institute. We calculated the weighted prevalence of ophthalmological manifestations, considering only the studies reporting the measurement of each manifestation.

**Results:**

Twenty-two articles (from 15 countries, published during 1994–2020) were included in the present systematic review. Ocular manifestations were observed in 85% of the studied pediatric and adolescent populations with DS. The most frequent ones were slanting fissures, oblique fissures, epicanthus, and epiblepharon.

**Conclusion:**

The ocular manifestations in the pediatric and adolescent populations with DS are varied, and some can irreversibly affect visual development. Screening of the pediatric population with DS should be conducted from the first months of age and continued annually.

**Systematic review registration:**

PROSPERO CRD42019127717

**Supplementary Information:**

The online version contains supplementary material available at 10.1186/s13643-022-01940-5.

## Introduction

DS is a chromosomal anomaly within the trisomy group characterized by an extra genetic material of chromosome 21. The most described mechanism is meiotic nondisjunction in 96% of cases. Other causes described include Robertsonian translocation, isochromosome, mosaicism, and partial trisomy [1, 2].

DS is one of the most frequent genetic disorders worldwide. It is estimated that the birth rate of children with DS is approximately 1 in 400–1,500 live births [3]. The prevalence of DS is estimated at 5.6 per 10,000 inhabitants in Europe [4] and 6.7 per 10,000 in the USA [5]. The prevalence of DS is greater in countries where abortion is illegal. In Latin America, from 1998 to 2005, the global rate of DS was 1.88%. Chile (2.47 per 10,000), Argentina (2.01), and Paraguay (1.98) have an above-the-average global rate of DS. Brazil (1.72), Colombia (1.72), Bolivia (1.55), Venezuela (1.49), Ecuador (1.48), and Uruguay (1.32) have a below-the-average global rate of DS [6].

Among the medical conditions presented in DS, the cognitive deficit is the most prevalent, followed by hearing deficits, hypothyroidism, congenital heart defects, and visual problems [7].

As is true for all chromosomal alterations, the drastic increase in gene dosage in DS can cause massive disarray of genes involved in organogenesis from the early stages of embryonic development. In DS, neural crest development and ectodermal alterations can be present. In the best-characterized mouse model of DS (Ts [1716] 65Dn mouse [hereafter Ts65Dn]), alterations in the development of the neural crest have been demonstrated as a common precursor of many structures affected in DS, such as the sensory organs [8]. Moreover, the pigmentation of the embryonic ophthalmic cup in trisomic animals is delayed compared to the normal ones [9]. Proteins encoded in several genes present in chromosome 21 are needed to inhibit angiogenesis, so specific manifestations, such as abnormal retinal and optic nerve angiogenesis in DS, can be explained [10]. Despite the high prevalence of DS, relatively few resources have been mobilized to support research into understanding its neurobiology [11].

Ophthalmologic manifestations are highly prevalent among the DS population, including manifestations that put visual development at risk. Refractive errors are common, especially hyperopia, which can lead to amblyopia in otherwise healthy eyes [12]. Studies have shown that, in individuals with cognitive impairment, having visual disabilities leads to a deficit in many daily aspects, such as social behavior, language abilities, adaptive behavior, communication skills, and autonomous living competencies [13]. All of them are aspects on which it is essential to intervene early to have an impact on the quality of life in these pediatric and adolescent populations.

This study aimed to systematically review the scientific evidence of ophthalmological and refractive findings of the pediatric and adolescent populations with DS and estimate the weighted prevalence of these findings and then generate recommendations regarding the ophthalmological evaluation and follow-up in this population.

## Materials and methods

### Protocol and registration

This study protocol was reported according to the Preferred Reporting Items for Systematic Reviews and Meta-Analysis Protocols (PRISMA-P) guidelines [14] (Supplementary material PRISMA-P checklists). The protocol registration can be found under the PROSPERO ID CRD42019127717.

### Eligibility criteria

We included all published observational studies with available abstract and original data in which the prevalence of ophthalmological manifestations was measured in the pediatric and adolescent populations with DS. Individuals between 0 and 21 years of age were included, since in the preliminary literature search, it was identified that some places consider the pediatric population up to 21 years of age in DS. The study was also included if the prevalence was not reported, but data for its calculation were available. Articles were excluded from the analysis if the study design was a review, letter to the editor, case report, case series, or systematic review and if the subjects presented ophthalmological manifestation secondary to therapies or conditions different from DS.

### Information sources

The literature search was conducted in the following electronic databases up to February 2020: MEDLINE, Cochrane Library, EMBASE, ScienceDirect, and LILACS. No limits regarding language and period of publication were used. The same search strategy was conducted in February 2021, including publications from 2020 to 2021.

As an example, MEDLINE search formula used was the following: ((((((((((down syndrome[MeSH Terms]) OR Down syndrome) OR Down's syndrome) OR trisomy 21[MeSH Terms]) OR Trisomy 21) OR partial trisomy 21 down syndrome[MeSH Terms]) OR partial trisomy 21) OR Mongolism)) AND (((((((((infant[MeSH Terms]) OR infant) OR child[MeSH Terms]) OR Child) OR Children) OR adolescent[MeSH Terms]) OR Adolescen*) OR teen*) OR Youth)) AND ((((((((((((((((((eye disease[MeSH Terms]) OR Eye*[Title/Abstract]) OR ophthalmology[MeSH Terms]) OR Ophthalm*[Title/Abstract]) OR Ocular[Title/Abstract]) OR Visual[Title/Abstract]) OR Vision[Title/Abstract]) OR Conjunctival[Title/Abstract]) OR Cornea*[Title/Abstract]) OR Lacrimal[Title/Abstract]) OR optic*[Title/Abstract]) OR orbit*[Title/Abstract]) OR Scleral[Title/Abstract]) OR Lens[Title/Abstract]) OR Pupil*[Title/Abstract]) OR Refractive[Title/Abstract]) OR Retina*[Title/Abstract]) OR Uveal[Title/Abstract]). The other search formulas can be found in Additional file 2.

### Search

We used a combination of exploded controlled vocabulary (MeSH, Emtree, DeCS) and free-text terms (e.g., spelling variants, plurals, synonyms, acronyms, and abbreviations) with field labels, truncation, proximity operators, and Boolean operators.

### Study selection

The electronic search was conducted by reviewers JMO, JDCS, and MBB. Duplicates were eliminated through a Zotero function and double-checked using an Excel function. Four reviewers conducted a critical reading of the title and abstract in a peer way to identify studies that potentially met the inclusion criteria outlined above. The disagreement between them was resolved by ophthalmology and neurodevelopment experts (CTG and ADLT).

### Data collection process

A data collection form was designed in Excel. Five authors (JMO, JDCS, MBB, IBA, and CTG) performed a peer review of the articles, and individual authors performed single data extractions.

### Data items

The variables extracted from each study included the author, study period, location, study design, number of children and adolescents with DS in each age interval, the prevalence of ocular manifestations, and refractive error definition used by the authors.

### Risk of bias in individual studies

We classified and assessed the articles according to their methodological designs, applying the checklists for analytical cross-sectional studies and prevalence studies of the Joanna Briggs Institute (JBI) to conduct this systematic review [15]. We used the toolkits provided within the checklists to assess the selection, performance, detection, attrition, and reporting bias. An expert methodologist (MGS) established cutting points for study inclusion: when 75% of major criteria were met for cross-sectional studies and when 66% of major criteria were met for prevalence studies (Additional file 1).

### Effect measures

To prevent overestimating and underestimating the prevalence of ocular manifestations in the pediatric and adolescent populations with DS, we only included data regarding specific ocular manifestations and did not extract information on grouped or overall ocular issues.

## Results

### Study selection

Our first search strategy extracted 3497 published articles (1318 from MEDLINE, 1215 from EMBASE, 855 from ScienceDirect, 92 from Cochrane Library, and 17 from LILACS). After removing duplicates, 3125 records were screened, 52 were evaluated through the JBI quality tools (Additional file 1), 52 full-text articles were assessed for eligibility, and 32 studies were excluded.

The search strategy was updated in February 2021, including publications from 2020 to 2021 (67 from MEDLINE, 1 from LILACS, 0 from Cochrane Library, 69 from EMBASE, and 14 in ScienceDirect). Only two articles met the inclusion criteria and were included in the present review [16, 17].

**Fig. 1:**
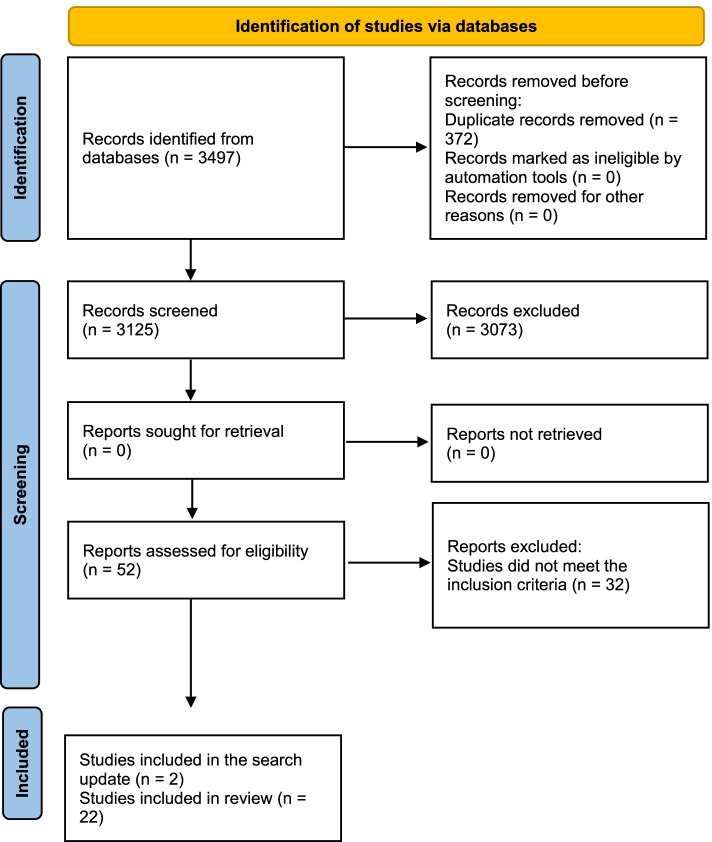
PRISMA 2020 flow diagram for new systematic reviews which included searches of databases and registers only

Finally, 22 articles met all inclusion criteria and were included in the present systematic review (Fig. 1).

### Studies' characteristics

Of the 22 published articles included in our review, only one was conducted in South America (Brazil), two in Africa (one from Egypt and one from Nigeria), two in North America (two from the USA), six in Europe (one from Croatia, one from Italy, one from Norway, one from Slovenia, and two from Spain), and 11 in Asia (one from China, three from Turkey, two from Japan, two from South Korea, two from Malaysia, and one from Iran). All studies were published between 1994 and 2020.

The studies included 44 to 546 participants. The pediatric and adolescent populations studied in the publications ranged from 0 to 19 years. Only one of the 22 studies did not include refraction under cycloplegia. Based on the study design, there were four comparative cross-sectional studies and 18 prevalence studies (Table 1).

**Table 1:** Characteristics and measures of the included studies

Author	Year	Location	Purpose	Design	N	DS diagnosis	Ages	Refraction under cycloplegic	Ametropia definition (D)
Afifi et al.	2013	Egypt	Prevalence of ophthalmological manifestations in children with DS	Prevalence	90	Karyotype	3 months to 10 years	Yes	Myopia: SE > −1.50 Hyperopia: SE > +1.00 Astigmatism: CD > 1.50
Akinci et al.	2009	Turkey	Prevalence of refractive errors, strabismus, nystagmus, and congenital cataract in the pediatric population with DS	Comparative cross-sectional	77	Clinical and genetic diagnosis	1 to 17 years	Yes	Myopia: SE > −0.50 Hyperopia: SE > +2.00 Astigmatism: CD > 1.00
Aslan et al.	2013	Turkey	Prevalence of preventable visual impairment in children with cognitive disability (including children with DS)	Comparative cross-sectional	90	Karyotype	4 to 12 years	Yes	Emmetropia: SE −1.00 to +1.00 Myopia: SE > −1.00 Hyperopia: SE > +1.00 Astigmatism: CD > 1.00
Fimiani et al.	2007	Italy	Prevalence of ophthalmological manifestations in children with DS	Prevalence	157	Karyotype	1 month to 18 years	Yes	Emmetropia: SE −0.75 to +0.75 Myopia: SE > −0.75 Hyperopia: SE > +0.75 Astigmatism: CD > 0.75
González-Viejo et al.	1996	Spain	Prevalence of ophthalmological manifestations in children with DS	Comparative cross-sectional	60	The pediatrician remitted the children with DS diagnosis	Mean 7.91, SD 4	Yes	ND
Haugen et al.	2001	Norway	Prevalence of strabismus and binocular function in children with DS	Prevalence	60	ND	3 months to 5 years	Yes	ND
Horio et al.	2018	Japan	Prevalence of refractive errors in children with DS	Prevalence	416	The pediatrician remitted the children with DS diagnosis	0 to 19 years	Yes	Emmetropia: −0.100 to +0.100 Myopia: Sph > −0.100 Hyperopia: Sph > +0.100 Astigmatism: CD > 0.100
Karlica et al.	2011	Croatia	Prevalence of ophthalmological manifestations in children with DS	Prevalence	153	Children belonged to a DS association	0 to 18 years	Yes	Myopia: Sph > 0.00 Hyperopia: Sph > 0.00 Astigmatism: CD > 0.00
Kim et al.	2002	South Korea	Prevalence of ophthalmological manifestations in children with DS	Prevalence	123	Karyotype	6 months to 14 years	Yes	Myopia: SE > −0.75 Hyperopia: SE > +0.75 Astigmatism: CD > 0.75
Kim et al.	2009	South Korea	Prevalence of refractive errors and strabismus in children with DS	Prevalence	148	ND	< 3 to 9 years	Yes	Emmetropia: −0.100 to +0.100 Myopia: Sph > −0.100 Hyperopia: Sph > +0.100 Astigmatism: CD > 0.100
Kranjc	2012	Slovenia	Prevalence of ophthalmological manifestations in children with DS	Prevalence	65	Karyotype	2 months to 13 years	Yes	Myopia: SE > −3.00 Hyperopia: SE> +3.00
Liza-Sharmini et al.	2006	Malaysia	Prevalence of ophthalmological manifestations in children with DS	Prevalence	60	Clinical and genetic diagnosis	0 to 17 years	Yes	Myopia: SE > −0.50 Hyperopia: SE > +0.50 Astigmatism: CD > 0.50
Makateb et al.	2019	Iran	Prevalence of ophthalmological manifestations in children with DS	Prevalence	182	Karyotype	10 to 20 years	Yes	Myopia: SE > −0.50 Hyperopia: SE > +0.50 Astigmatism: CD > 0.50
Mohd-Ali et al.	2006	Malaysia	Prevalence of refractive errors and strabismus in children with DS	Prevalence	73	ND	1 to 12 years	Yes	Emmetropia: SE +2.00 to −0.25 Astigmatism: CD > 1.00
Nogueira-Pires-da-Cunha et al.	1995	Brazil	Prevalence of ophthalmological manifestations in children with DS	Prevalence	152	Karyotype	0 to 18 years	Yes	Emmetropia: SE −0.50 to −0.50 Myopia: SE > −0.50 Hyperopia: SE > +0.50 Astigmatism: SE > 0.50
Nwokedi et al.	2018	Nigeria	Prevalence of refractive errors in children with DS	Prevalence	91	Children belonged to a DS foundation	5 to 18 years	No	Myopia: SE > −0.75 Hyperopia: SE > +0.75 Astigmatism: CD > 0.75
Puig et al.	2002	Spain	Prevalence of ametropia and strabismus in children with DS	Prevalence	546	Children belonged to a DS foundation	1 month to 18 years	Yes	Myopia: SE > −0.00 Hyperopia: SE > +0.75 Astigmatism: CD > 2.00
Roizen et al.	1994	USA	Prevalence of ophthalmological manifestations in children with DS	Prevalence	77	The pediatrician remitted the children with DS diagnosis	2 months to 19 years	Yes	< 3 y.o hyperopia if > +3.00 Astigmatism: CD > 1.5
Terai et al.	2018	Japan	Prevalence of ophthalmological manifestations in children with DS	Prevalence	222	Karyotype	3 months to 19 years	Yes	Myopia: Sph > −0.100 Hyperopia: Sph > +0.100 Astigmatism: CD > 0.100
Tsiaras et al.	1999	USA	Prevalence of ophthalmological manifestations in children with DS	Prevalence	68	Karyotype	5 to 19 years	Yes	Hyperopia: SE > +4.00 Astigmatism: CD > 1.75
Ugurlu et al.	2020	Turkey	Prevalence of ophthalmological manifestations in children with DS	Comparative cross-sectional	44	ND	7 to 18 years	Yes	Astigmatism: CD > 1.00
Wong et al.	1997	China	Prevalence of ophthalmological manifestations in children with DS	Prevalence	140	Karyotype	3 months to 13 years	Yes	ND

*SE* spherical equivalent, *CD* cylindric diopter, *ND* no data

### Risk of bias within studies

Of the 52 studies evaluated in full text through the JBI critical appraisal tools (Additional file 1), 11 did not meet minimum quality criteria and were excluded. The 22 studies included in the review met the necessary quality criteria (Table 2).

**Table 2:** JBI quality tools result for final selected articles

Author	Tool	Quality results (%)
Afifi et al.	Prevalence	8/9 (88%)
Akinci et al.	Comparative cross-sectional	7/8 (87%)
Aslan et al.	Comparative cross-sectional	7/8 (87%)
Fimiani et al.	Prevalence	8/9 (88%)
González-Viejo et al.	Comparative cross-sectional	7/8 (87%)
Haugen et al.	Prevalence	8/9 (88%)
Horio et al.	Prevalence	8/9 (88%)
Karlica et al.	Prevalence	9/9 (100%)
Kim et al.	Prevalence	8/9 (88%)
Kim et al.	Prevalence	8/9 (88%)
Kranjc	Prevalence	8/9 (88%)
Liza-Sharmini et al.	Prevalence	8/9 (88%)
Makateb et al.	Prevalence	8/9 (88%)
Mohd-Ali et al.	Prevalence	7/9 (77%)
Nogueira-Pires-da-Cunha et al.	Prevalence	8/9 (88%)
Nwokedi et al.	Prevalence	7/9 (77%)
Puig et al.	Prevalence	9/9 (100%)
Roizen et al.	Prevalence	8/9 (88%)
Terai et al.	Prevalence	8/9 (88%)
Tsiaras et al.	Prevalence	8/9 (88%)
Ugurlu et al.	Comparative cross-sectional	7/8 (87%)
Wong et al.	Prevalence	8/9 (88%)

### Synthesis of results

The presence of ocular manifestations was observed in 85% of the studied pediatric and adolescent populations with DS. This value was found, considering only studies that reported the percentage of ocular manifestations in the DS group. These studies were conducted by Afifi et al. [18], Akinci et al. [19], Fimiani et al. [20], Kim et al. [21], Roizen et al. [22], Terai et al. [23], and Wong et al. [24].

The most frequent ocular manifestations (prevalence > 70%) were slanting fissures, oblique fissure, epicanthus, and epiblepharon. Less frequent ocular manifestations (< 1%) included intermittent exotropia, microcornea, tilted disk, myopic chorioretinitis, retinal vessel tortuosity, preretinal hemorrhage, microphthalmos, myelinated nerve fiber, and keratoconus. Moreover, the most frequent spherical refractive defect was hyperopia (Table 3).

**Table 3:** Prevalence of ocular manifestations in children with DS

	Ocular manifestations	***n***	Sample	Prevalence
**Refraction**	Emmetropia	233	1078	21.61%
	Anisometropia	360	1407	25.59%
	Myopia	534	2482	21.51%
	Hyperopia	727	1996	36.42%
	Astigmatism	922	2480	37.18%
	Mixed refractive error	75	140	53.57%^a^
	Antimetropia	2	91	2.19%^a^
	Amblyopia	66	498	13.25%
	Low vision best eye (CF or less)	9	265	3.40%
**Eye movements and ocular alignment**	Nystagmus	318	2041	15.58%
	Strabismus	775	2311	33.54%
	Esotropia	542	1955	27.72%
	Intermittent esotropia	19	138	13.77%
	Exotropia	85	1815	4.68%
	Intermittent exotropia	1	138	0.72%
	Vertical deviation	82	1131	7.25%
	Abnormal head posture	53	966	5.49%
**Eyelid and annexa**	Ptosis	10	392	2.55%
	Oblique fissure	196	225	87.11%
	Slanting fissures	436	483	90.27%
	Epicanthus	779	1005	77.51%
	Epiblepharon	289	405	71.36%
	Nasolacrimal duct obstruction	154	1063	14.49%
	Blepharitis	219	515	42.52%
	Conjunctivitis	4	60	6.67%
	Blepharoconjunctivitis	120	852	14.08%
	Epiphora	14	65	21.54%
	Entropion	1	60	1.67%
	Chalazion	3	217	1.38%
	Floppy eyelid	33	182	18.13%
	Stye	2	60	3.33%
**Cornea**	Keratoconus	4	916	0.44%
	Superficial punctate keratitis	58	123	47.15%^a^
	Corneal leukoma	4	365	1.10%
	Microcornea	1	157	0.64%
**Iris**	Iris abnormalities	83	424	19.58%
	Brushfield spots	23	654	3.52%
**Lens**	Cataract	191	1760	10.85%
**Optic nerve**	Glaucoma	11	925	1.19%
	Hypoplastic fovea	6	65	9.23%^a^
	Supernumerary optic disk vessels	37	153	24.18%^a^
	Optic nerve dysplasia	2	90	2.22%^a^
	Optic disk pallor	6	222	2.70%
	Tilted disc	1	157	0.63%^a^
	Peripapillary vascular abnormalities	13	222	5.85%^a^
	Pseudopapilledema	2	77	2.60%
**Retina**	Retinal pigment epithelium hyperplasia	6	188	3.19%
	Tigroid fundus	80	222	36.03%^a^
	Retinal detachment	2	137	1.46%
	Retinal vessel abnormalities	29	188	15.43%
	Retinal abnormalities	52	309	16.83%
	Myelinated nerve fiber	1	222	0.45%^a^
	Retinoblastoma	1	60	1.66%^a^
	Myopic chorioretinitis	1	157	0.63%^a^
	Retinal vessel tortuosity	1	157	0.63%^a^
	Preretinal hemorrhage	1	157	0.63%^a^
**Uvea**	Uveitis	1	60	1.66%^a^
	Chorioretinal coloboma	1	77	1.29%^a^
**Other**	Microphthalmos	1	157	0.63%^a^

^a^Calculation made on the results of a single study that reports this manifestation

Some ocular manifestations were only described in one study; therefore, this prevalence is based on the sample of each publication. Some variables were grouped to determine a more accurate prevalence of the manifestation. For example, the vertical deviation variable includes cases of hypertropia and hypotropia, the abnormal head posture variable includes head tilt and head turn, and the cataract variable includes lens opacities (Table 3).

The age range of the pediatric and adolescent populations was 0 to 20 years. To compare the age groups, studies with results grouped in similar age ranges and the same outcome measured were compared, obtaining four age groups: group 1, < 1 year (one study); group 2, < 5 years (five studies); group 3, 6–12 years (two studies); and group 4, > 12 years (two studies) (Table 4).

**Table 4:** Analysis of ophthalmological manifestations of children with DS by age

Author	Group 1		Group 2						Group 3			Group 4		
	Afifi et al.	Total	Horio et al.	Terai et al.	Liza-Sharmini et al.	Nogueira-Pires da Cunha et al.	Afifi et al.	Total	Aslan et al.	Nogueira-Pires da Cunha et al.	Total	Nogueira-Pires da Cunha et al.	Horio et al.	Total
Ages	< 1 years		≤ 6 years	0 to < 4 years	0 to < 5 years	0 to 4 years	1 to 5 years		4 to 12 years	5 to 11 years		12 to 18 years	13 to 19 years	
Cycloplegia	Yes		Yes	Yes	Yes	Yes	Yes		Yes	Yes		Yes	Yes	
**N**	35	35	277	204	60	84	46	671	90	57	147	11	29	40
**Emmetropia**	-	-	71	43	-	-	-	24%	35	-	39%	-	9	31%
**Anisometropia**	-	-	-	-	-	-	-	-	3	-	3%	-	-	-
**Myopia**	2	6%	62	23	4	10	5	15%	20	7	18%	2	16	45%
**Hyperopia**	6	17%	144	248	4	30	8	**65%**	33	7	27%	2	4	15%
**Astigmatism**	2	6%	238	239	0	42	8	**79%**	16	42	39%	7	27	**85%**
**Amblyopia**	-	-	-	-	-	18	-	21%	-	8	14%	3	-	27%
**Oblique fissure**	-	-	-	-	-	65	-	**77%**	-	51	**89%**	9	-	**82%**
**Iris abnormalities**	-	-	-	-	-	33	-	39%	-	37	**65%**	9	-	**82%**
**Epicanthus**	-	-	-	-	-	53	-	63%	-	35	61%	4	-	36%
**Nystagmus**	1	3%	-	-	-	17	2	15%	7	7	10%	4	-	36%
**Strabismus**	4	11%	-	-	-	27	7	26%	30	21	35%	9	-	**82%**
**Esotropia**	-	-	-	-	-	-	-	-	21	-	23%	-	-	-
**Exotropia**	-	-	-	-	-	-	-	-	4	-	4%	-	-	-
**Cataract**	0	0%	-	-	-	2	4	5%	7	10	12%	8	-	**73%**
**Nasolacrimal duct obstruction**	11	**31%**	-	-	-	29	12	32%	-	14	25%	3	-	27%
**Optic nerve dysplasia**	1	3%	-	-	-	-	0	0%	-	-		-	-	-
**Blepharitis**	-	-	-	-	-	24	-	29%	-	18	32%	3	-	27%
**Blepharoconjunctivitis**	11	**31%**	-	-	-	-	12	26%	-	-		-	-	-
**Retinal abnormalities**	-	-	-	-	-	22	-	26%	-	17	30%	3	-	27%
**Vertical deviation**	-	-	-	-	-	-	-	-	7	-	8%	-	-	-

In group 1, the most frequent ocular findings were nasolacrimal duct obstruction and blepharoconjunctivitis. In group 2, astigmatism, oblique palpebral fissure, and hyperopia were the most frequent manifestations. In group 3, oblique fissures and iris abnormalities were the most relevant characteristics. Moreover, in group 4, astigmatism, iris abnormalities, oblique eyelid fissures, and cataracts were the most frequent abnormalities (Table 4).

## Discussion

As previously described, DS is one of the most prevalent genetic syndromes globally [3]. However, the number of publications about ophthalmological manifestations in the pediatric population with DS in our region, South America, is low. There is a need to perform these studies in our region to generate clinical recommendations based on data from the Latin American population.

In the present study, a high weighted prevalence (85%) of ophthalmological manifestations was found in the pediatric and adolescent populations with DS. The lowest prevalence was found in the study by Afifi et al. [18] (57%), and the highest prevalence was found in the study by Fimiani et al. [20], where 100% of the children evaluated presented ophthalmological manifestations.

The most prevalent ocular manifestations found in our study were slanting fissures, oblique fissure, epicanthus, and epiblepharon. This result corresponds to widely described abnormalities within the DS phenotype-genotype. In a study that compared children and young individuals with DS in different countries (Africans, Asians, and Latin Americans) through digital facial analysis technology, it was shown that the most common feature found was upslanted palpebral fissures, and specifically, epicanthal folds in Asians were found in 71% [25].

Regarding other ophthalmological manifestations, a low vision was observed in a small percentage of children and adolescents. Inflammation of the ocular adnexa may be partly associated with the disposition of the eyelids and anatomical changes, such as oblique fissures, slanting fissures, and epicanthus. It must be treated to prevent more severe complications.

Brushfield spots at the iris have always been a well-known finding in children and adolescents with DS; however, the present study showed a low prevalence. Nevertheless, many studies named “iris abnormalities” in their texts. It would be convenient to know semiologically the types of abnormalities they refer to classify this manifestation correctly. The same occurs with the finding “retinal vessel abnormalities,” a manifestation with a frequency > 15% in our study. Still, as previously mentioned, the abnormality must be correctly expressed to understand its implications for the patient.

At the lens, cataract was presented in 10.85% of patients. The study by Liza-Sharmini A. T. et al. classified cataracts as congenital, developmental, and secondary; however, all the patients found with this anomaly were congenital [26]. In a study conducted in the UK, among children with congenital or infantile cataracts diagnosed during the first year, 5.4% were DS patients, with 61.5% being diagnosed in the neonatal period [27]. B Haargaard et al. estimated frequency of early cataracts in DS cases at 1.4% [28].

In the specific case of keratoconus, we consider that, although a high prevalence has been described in different studies of individuals with DS, ranging from 32 [29] to 38.8% [30], in the present review, as we only considered complete ophthalmological assessment and diagnosis of keratoconus are only through corneal topography, we advise interpreting this result with caution.

Given the prevalence of refractive errors and strabismus, we consider these conditions need to be assessed and treated during the critical period of visual pathway development. A longitudinal study by Cregg et al. showed that refractive errors are not always present in early infancy; however, there is a high prevalence of strabismus, which cannot be attributed to the presence of hyperopia or anisometropia [31]. Conversely, the study by Haugen et al. reported that refractive errors in children with DS are close to those in general children at birth, and differentiation from the general population occurs after two years of age. The authors consider that the emmetropization process is absent in many children with DS, as many remain with stable hyperopia or even develop increasing hyperopia over the years [32].

Hypo-accommodation in children with DS is frequent and has been widely studied; however, its cause is still unknown. This deficit could involve differences in the accommodative/convergence relationship, which explains the high prevalence found of strabismus (33.54%), exotropia (4.68%), and esotropia (27.72%). A theory also proposes that the thinner crystalline lens found in DS children limits the change in shape and power increase to provide an appropriate accommodative response; however, this has not been proven experimentally [33].

Different recommendations exist regarding the ophthalmological screening in pediatric and adolescent populations with DS. The American Academy of Pediatrics recommends this evaluation in the first 6 months of life. Children aged between 3 and 5 years must be checked by an ophthalmologist every 1 or 2 years, and children aged from 5 to 13 years every 2 years [34]. Haugen et al. suggest the first examination at 1 month of age, then at 1 year of age, at 2–3 years of age, at 5–6 years of age, and after that, every 5 years. In case of positive findings, the ophthalmologist should determine the examination frequency individually [35]. Recently, Robinson et al. recommended first ophthalmological and orthoptic examination between 6 and 12 months of age, followed by examinations every 3–6 months for children under 2 years of age, every 6 months for children aged 2–5 years, annually for children 5–10 years of age, and, after that, to be decided on an individual basis [36].

With the results of our study about manifestations according to age groups, we consider that, it is essential to conduct screening to evaluate pathologies, such as congenital cataracts, and treat them early in the first month of life. Later in the escolar age, puberty, and adolescence, we consider that annual controls are required to evaluate the refractive and alignment errors that can generate amblyopia early and later aggravate the cognitive disorder due to low vision.

### Limitations

In the present study, there could be an overestimation bias of the global prevalence of ophthalmological manifestations, given that some included studies have a risk of selection bias because the patients were evaluated in the ophthalmological consultation due to the presence of ocular or visual alterations. Conversely, although we rigorously searched each publication to see how the pediatric population had been diagnosed with DS, some articles did not include this information, even though they assured the children had the diagnosis. Moreover, a limitation of this study was the existence of multiple definitions for the refractive errors, which could generate an overestimation or underestimation of these findings.

### Recommendation

Global recommendations for screening examinations in patients with DS should be established. In future research, unified concepts should be used to define the ranges of refractive errors to compare the results of the studies.

## Conclusions

DS is a highly prevalent chromosomopathy worldwide. We found a weighted prevalence of ophthalmological manifestations of 85% in the pediatric and adolescent populations with DS. The ophthalmological features varied according to age. In newborns, the most common findings are associated with soft tissue and adnexa inflammation, and in scholar years, the most frequent manifestations are related to refractive errors. We consider that screening of the pediatric population with DS should be conducted from the first months of age and continue annually to determine the need for treatment of amblyopia causes, such as strabismus, media opacities, and refractive errors, in this way preventing irreversible visual development alterations

## Supplementary Information

**Additional file 1.**

**Additional file 2.**

**Additional file 3.** PRISMA 2020 Checklist.

## Data Availability

All data generated or analyzed during this study are included in this published article and available from the corresponding author on reasonable request.

## Abbreviations

DS: Down syndrome
PRISMA-P: Preferred Reporting Items for Systematic Reviews and Meta-Analysis Protocols
MeSH: Medical Subject Headings
Emtree: Embase subject headings
DeCS: Descriptores en Ciencias de la Salud
JMO: Juliana Muñoz-Ortiz
JDCS: Jesús David Charry-Sánchez
IBA: Isabella Bechara-Arango
MBB: Mariana Blanco-Becerra
CTG: Claudia Talero-Gutiérrez
MGS: Marcela Gomez-Suarez
ADLT: Alejandra de-la-Torre
JBI: Joanna Briggs Institute

## References

[1] Down Syndrome - StatPearls - NCBI Bookshelf (n.d.). https://www.ncbi.nlm.nih.gov/books/NBK526016/. Accessed 17 Jan 2021.

[2] Claret-Torrents C, Goday-Arno A, Cerdà-Esteve M, Flores-Le Roux J, Chillarón-Jordan JJ, Cano-Pérez JF (2009). Hyperthyroidism in Down syndrome. Int Med Rev Down Syndr.

[3] Kazemi M, Salehi M, Kheirollahi M (2016). Down syndrome: current status, challenges and future perspectives. Int J Mol Cell Med.

[4] de Graaf G, Buckley F, Skotko BG. Estimation of the number of people with Down syndrome in Europe. Eur J Hum Genet. 2020;1–9. 10.1038/s41431-020-00748-y. 10.1038/s41431-020-00748-y PMC7940428 33130823

[5] De Graaf G, Buckley F, Skotko BG (2017). Estimation of the number of people with Down syndrome in the United States. Genet Med..

[6] Nazer J, Cifuentes L (2011). Estudio epidemiológico global del síndrome de Down, Revista Chilena de. Pediatría..

[7] Roizen NJ, Magyar CI, Kuschner ES, Sulkes SB, Druschel C, van Wijngaarden E, Rodgers L, Diehl A, Lowry R, Hyman SL (2014). A community cross-sectional survey of medical problems in 440 children with Down syndrome in New York State. J Pediatr.

[8] Roper RJ, VanHorn JF, Cain CC, Reeves RH (2009). A neural crest deficit in Down syndrome mice is associated with deficient mitotic response to Sonic hedgehog. Mech Dev.

[9] Ludwig M, Busch L, Winking H (1997). The embryonic development of sensory organs and the skull in the trisomy 16 mouse, an animal model for Down’s syndrome. Ann Anat.

[10] Parsa C, Almer Z (2008). Supranumerary optic disc vessels may indicate reduced systemic angiogenesis in Down syndrome. Br J Ophthalmol.

[11] Gardiner K, Herault Y, Lott IT, Antonarakis SE, Reeves RH, Dierssen M (2010). Down syndrome: from understanding the neurobiology to therapy. J Neurosci.

[12] da Cunha RP, Moreira JB (1996). Ocular findings in Down’s syndrome. Am J Ophthalmol.

[13] Evenhuis HM, Sjoukes L, Koot HM, Kooijman AC (2009). Does visual impairment lead to additional disability in adults with intellectual disabilities?. J Intellect Disabil Res.

[14] Moher D, Shamseer L, Clarke M, Ghersi D, Liberati A, Petticrew M, Shekelle P, Stewart LA (2015). Preferred reporting items for systematic review and meta-analysis protocols (PRISMA-P) 2015 statement. Syst Rev.

[15] Munn Z, Moola S, Lisy K, Riitano D, Tufanaru C (2015). Methodological guidance for systematic reviews of observational epidemiological studies reporting prevalence and cumulative incidence data. Int J Evid Based Healthcare.

[16] Makateb A, Hashemi H, Farahi A, Mehravaran S, Khabazkhoob M, Asgari S (2020). Ocular alignment, media, and eyelid disorders in Down syndrome. Strabismus..

[17] Ugurlu A, Altinkurt E. Ophthalmologic manifestations and retinal findings in children with down syndrome. J Ophthalmol. 2020;2020:9726261. 10.1155/2020/9726261. 10.1155/2020/9726261 PMC7029299 32089873

[18] Afifi HH, Abdel Azeem AA, El-Bassyouni HT, Gheith ME, Rizk A, Bateman JB (2013). Distinct ocular expression in infants and children with Down syndrome in Cairo, Egypt: myopia and heart disease. JAMA Ophthalmol.

[19] Akinci A, Oner O, Bozkurt OH, Guven A, Degerliyurt A, Munir K (2009). Refractive errors and strabismus in children with down syndrome: a controlled study. J Pediatr Ophthalmol Strabismus.

[20] Fimiani F, Iovine A, Carelli R, Pansini M, Sebastio G, Magli A (2007). Incidence of ocular pathologies in Italian children with Down syndrome. Eur J Ophthalmol.

[21] Kim JH, Hwang J-M, Kim HJ, Yu YS (2002). Characteristic ocular findings in Asian children with Down syndrome. Eye (Lond).

[22] Roizen NJ, Mets MB, Blondis TA (1994). Ophthalmic disorders in children with Down syndrome. Dev Med Child Neurol.

[23] Terai T, Eda S, Sugasawa J, Tonari M, Matsuo J, Oku H, Ikeda T (2018). Ocular findings in Japanese children with Down syndrome: the course of visual acuity and refraction, and systemic and ocular anomalies. Clin Ophthalmol.

[24] Wong V, Ho D (1997). Ocular abnormalities in Down syndrome: an analysis of 140 Chinese children. Pediatr Neurol.

[25] Kruszka P, Porras AR, Sobering AK, Ikolo FA, La Qua S, Shotelersuk V, Chung BH, Mok GT, Uwineza A, Mutesa L (2017). Down syndrome in diverse populations. Am J Med Genet Part A.

[26] Liza-Sharmini AT, Azlan ZN, Zilfalil BA. Ocular findings in Malaysian children with Down syndrome, Singapore. Med J. 2006;47:14–9. https://www.sma.org.sg/smj/4701/4701a1.pdf. 16397715

[27] Rahi JS, Dezateux C (2000). British Congenital Cataract Interest Group, Congenital and infantile cataract in the United Kingdom: underlying or associated factors. Invest Ophthalmol Vis Sci.

[28] Haargaard B, Fledelius HC (2006). Down’s syndrome and early cataract. Br J Ophthalmol.

[29] Imbornoni LM, Wise RE, Taravella MJ, Hickey F, McCourt EA (2020). Keratoconus and corneal morphology in patients with Down syndrome at a pediatric hospital. JAAPOS..

[30] Mathan JJ, Gokul A, Simkin SK, Meyer JJ, Patel DV, McGhee CN (2020). Topographic screening reveals keratoconus to be extremely common in Down syndrome. Clin Exp Ophthalmol..

[31] Cregg M, Woodhouse JM, Stewart RE, Pakeman VH, Bromham NR, Gunter HL, Trojanowska L, Parker M, Fraser WI (2003). Development of refractive error and strabismus in children with Down syndrome. Invest Ophthalmol Vis Sci.

[32] Haugen OH, Hovding G, Lundstrom I (2001). Refractive development in children with Down’s syndrome: a population based, longitudinal study. Br J Ophthalmol.

[33] Little J-A (2015). Accommodation deficit in children with Down syndrome: practical considerations for the optometrist. Clin Optom.

[34] Bull MJ; Committee on Genetics (2011). Health supervision for children with Down syndrome. Pediatrics..

[35] Haugen OH, Høvding G, Riise R (2004). Øyeforandringer ved Downs syndrom. Tidsskr Nor Lægeforen..

[36] Robinson J, Pompe MT, Gerth-Kahlert C. Challenges in patients with trisomy 21: a review of current knowledge and recommendations. J Ophthalmol. 2021;2021. 10.1155/2021/8870680 PMC8172292 34123415

