# Supplementary material for: Prevalence of ophthalmological manifestations in pediatric and adolescent populations with Down syndrome: a systematic review of the literature
Source: Syst Rev. 2022 Apr 22;11:75. doi: 10.1186/s13643-022-01940-5 (PMC9027460; doi:10.1186/s13643-022-01940-5)
Supplement: Supplementary file 1 — Additional file 1. [file 13643_2022_1940_MOESM1_ESM.pdf]

JBI CRITICAL APPRAISAL CHECKLIST FOR ANALYTICAL CROSS SECTIONAL STUDIES

Reviewer______________________________________ Date______________________________

Author_______________________________________Year_________ Record Number_________

	Yes	No	Unclear	Not applicable
1. Were the criteria for inclusion in the sample clearly defined?	☑	☐	☐	☐
2. Were the study subjects and the setting described in detail?	☑	☐	☐	☐
3. Was the exposure measured in a valid and reliable way?	☑	☐	☐	☐
4. Were objective, standard criteria used for measurement of the condition?	☑	☐	☐	☐
5. Were confounding factors identified?	☑	☐	☐	☐
6. Were strategies to deal with confounding factors stated?	☑	☐	☐	☐
7. Were the outcomes measured in a valid and reliable way?	☑	☐	☐	☐
8. Was appropriate statistical analysis used?	☑	☐	☐	☐

Overall appraisal: Include ☐ Exclude ☐ Seek further info ☐

Comments (Including reason for exclusion)

Major criteria ✓

Minor criteria ✓

Inclusion criteria: 6/8 (At least 5 of major criteria)

© JBI, 2020. All rights reserved. JBI grants use of these tools for research purposes only. All other enquiries should be sent to jbisynthesis@adelaide.edu.au.

JBI CRITICAL APPRAISAL CHECKLIST FOR STUDIES REPORTING PREVALENCE DATA

Reviewer______________________________________ Date______________________________

Author_______________________________________Year_________ Record Number_________

	Yes	No	Unclear	Not applicable
1. Was the sample frame appropriate to address the target population?	☑	☐	☐	☐
2. Were study participants sampled in an appropriate way?	☑	☐	☐	☐
3. Was the sample size adequate?	☑	☐	☐	☐
4. Were the study subjects and the setting described in detail?	☑	☐	☐	☐
5. Was the data analysis conducted with sufficient coverage of the identified sample?	☑	☐	☐	☐
6. Were valid methods used for the identification of the condition?	☑	☐	☐	☐
7. Was the condition measured in a standard, reliable way for all participants?	☑	☐	☐	☐
8. Was there appropriate statistical analysis?	☑	☐	☐	☐
9. Was the response rate adequate, and if not, was the low response rate managed appropriately?	☑	☐	☐	☐

Overall appraisal: Include ☐ Exclude ☐ Seek further info ☐

Comments (Including reason for exclusion)

Major criteria ✓

Minor criteria ✓

Inclusion criteria: 6/9 (At least 5 of major criteria)

© JBI, 2020. All rights reserved. JBI grants use of these tools for research purposes only. All other enquiries should be sent to jbisynthesis@adelaide.edu.au.
