# Supplementary material for: Prevalence of ophthalmological manifestations in pediatric and adolescent populations with Down syndrome: a systematic review of the literature
Source: Syst Rev. 2022 Apr 22;11:75. doi: 10.1186/s13643-022-01940-5 (PMC9027460; doi:10.1186/s13643-022-01940-5)
Supplement: Supplementary file 2 — Additional file 2. [file 13643_2022_1940_MOESM2_ESM.pdf]

PUBMED

(((((((((down syndrome[MeSH Terms]) OR Down syndrome) OR Down's syndrome) OR trisomy 21[MeSH Terms]) OR Trisomy 21) OR partial trisomy 21 down syndrome[MeSH Terms]) OR partial trisomy 21) OR Mongolism)) AND ((((((((infant[MeSH Terms]) OR infant) OR child[MeSH Terms]) OR Child$) OR Children) OR adolescent[MeSH Terms]) OR Adolescen*) OR teen*) OR Youth$)) AND ((((((((((((((((eye disease[MeSH Terms]) OR Eye[Title/Abstract]) OR ophthalmology[MeSH Terms]) OR Ophthalm*[Title/Abstract]) OR Ocular[Title/Abstract]) OR Visual[Title/Abstract]) OR Vision[Title/Abstract]) OR Conjunctival[Title/Abstract]) OR Cornea*[Title/Abstract]) OR Lacrimal[Title/Abstract]) OR optic*[Title/Abstract]) OR orbit*[Title/Abstract]) OR Scleral[Title/Abstract]) OR Lens[Title/Abstract]) OR Pupil*[Title/Abstract]) OR Refractive[Title/Abstract]) OR Retina*[Title/Abstract]) OR Uveal[Title/Abstract])*

EMBASE

('down syndrome'/exp OR 'down syndrome' OR 'down`s syndrome' OR 'downs syndrome' OR 'down disease' OR 'idiocy, mongolian' OR 'langdon down disease' OR 'langdon down syndrome' OR 'mongolian idiocy' OR 'mongolism' OR 'mongoloid idiocy' OR 'mongoloidism' OR 'translocation 15 21 22' OR 'trisomy 21 syndrome') AND ('child'/exp OR 'child' OR 'children' OR 'infant'/exp OR 'infant' OR 'adolescent'/exp OR 'adolescent' OR 'teenager') AND ('ophthalmology'/exp OR 'ophthalmologic examination' OR 'ophthalmology' OR 'space ophthalmology' OR 'eye disease'/exp OR 'disease, eye' OR 'eye disease' OR 'eye diseases' OR 'eye diseases, hereditary' OR 'eye disorder' OR 'eye manifestations' OR 'hereditary eye disease' OR 'neovascularisation, eye' OR 'neovascularization, eye' OR 'ocular abnormalities' OR 'ocular abnormality' OR 'ocular disease' OR 'ocular disorder' OR 'ocular disturbance' OR 'oculopathy' OR 'ophthalmopathology' OR 'ophthalmopathy')

COCHRANE

#1	*MeSH descriptor: [Down Syndrome] explode all trees*	*331*
#2	*Down syndrome*	*1930*
#3	*Trisomy 21*	*165*
#4	*MeSH descriptor: [Ophthalmology] explode all trees*	*118*
#5	*ophthalmology*	*16607*
#6	*Ocular*	*11942*
#7	*Eye*	*24511*
#8	*MeSH descriptor: [Eye Diseases] explode all trees*	*17132*
#9	*#1 OR #2 OR #3*	*2022*
#10	*#4 OR #5 OR #6 OR #7*	*36328*
#11	*MeSH descriptor: [Pediatrics] explode all trees*	*620*
#12	*MeSH descriptor: [Infant] explode all trees*	*15136*
#13	*MeSH descriptor: [Child] explode all trees*	*1125*
#14	*MeSH descriptor: [Adolescent] explode all trees*	*98684*
#15	*Pediatric**	*50498*
#16	*infant$*	*42717*
#17	*Child**	*135996*
#18	*Adolescent$*	*119837*
#19	*#11 OR #12 OR #13 OR #14 OR #15 OR #16 OR #17 OR #18*	*231940*
#20	*#9 AND #10 AND #19*	*71*

LILACS

Síndrome de Down [Descriptor de asunto] and oftalmopatías [Descriptor de asunto

SPRINGERLINK

(down syndrome OR trisomy 21 OR Mongolism OR 47,XY,+21 OR Trisomy G OR 47,XX,+21 OR Down's Syndrome OR Downs Syndrome) AND (eye OR ocular manifestations OR Eye Manifestations OR vision OR visual defects)

Limits
Conference Paper
Chapter

PROQUEST

down syndrome OR trisomy 21 OR Mongolism OR 47,XY,+21 OR Trisomy G OR 47,XX,+21 OR Down's Syndrome OR Downs Syndrome) AND (eye OR ocular manifestations OR Eye Manifestations OR vision OR visual defects)

Limits
Humans

Age group
Adolescent (13-18 years), Baby (from 1 to 23 months), Child (from 6 to 12 years old), Preschooler (from 2 to 5 years old)

Type of source
Blogs, podcasts and websites, Reports, Books, Other sources, Newspapers, General magazines, Professional journals, Press services, Doctoral theses and dissertations, Audio and video works
